# Different lipid profiles according to ethnicity in the Heart of Soweto study cohort of *de novo* presentations of heart disease

**DOI:** 10.5830/CVJA-2012-036

**Published:** 2012-08

**Authors:** Karen Sliwa, Sandrine Lecour, Melinda J Carrington, Simon Stewart, Jasmine G Lyons, Simon Stewart, A David Marais, Frederick J Raal

**Affiliations:** Hatter Institute for Cardiovascular Research in Africa and IIDMM, Faculty of Health Sciences, University of Cape Town, South Africa; Soweto Cardiovascular Research Unit, Chris Hani Baragwanath Hospital, University of the Witwatersrand, Johannesburg, South Africa; Hatter Institute for Cardiovascular Research in Africa and IIDMM, Faculty of Health Sciences, University of Cape Town, South Africa; Soweto Cardiovascular Research Unit, Chris Hani Baragwanath Hospital, University of the Witwatersrand, Johannesburg, South Africa; Baker IDI Heart and Diabetes Institute, Melbourne, Australia; Soweto Cardiovascular Research Unit, Chris Hani Baragwanath Hospital, University of the Witwatersrand, Johannesburg, South Africa; Baker IDI Heart and Diabetes Institute, Melbourne, Australia; Baker IDI Heart and Diabetes Institute, Melbourne, Australia; Department of Internal Medicine, Groote Schuur Hospital, Observatory, Cape Town, South Africa; Carbohydrate and Lipid Metabolism Research Unit, University of the Witwatersrand, Johannesburg, South Africa

**Keywords:** Africa, heart disease, lipids, ethnicity/race, epidemiologic transition

## Abstract

**Background:**

Historically, sub-Saharan Africa has reported low levels of atherosclerotic cardiovascular disease (CVD). However as these populations undergo epidemiological transition, this may change.

**Methods:**

This was an observational cohort study performed at Chris Hani Baragwanath Hospital in Soweto, South Africa. As part of the Heart of Soweto study, a clinical registry captured detailed clinical data on all *de novo* cases of structural and functional heart disease presenting to the Cardiology unit during the period 2006 to 2008. We examined fasting lipid profiles in 2 182 patients (of 5 328 total cases) according to self-reported ethnicity. The study cohort comprised 1 823 patients of African descent (61% female, aged 56 ± 16 years), 142 white Europeans (36% female, aged 57 ± 13 years), 133 Indians (51% female, aged 59 ± 12 years) and 87 of mixed ancestry (40% female, aged 56 ± 12 years).

**Results:**

Consistent with different patterns in heart disease aetiology, there were clear differences in total cholesterol (TC), low-density lipoprotein cholesterol (LDL-C) and triglycerides across ethnicities (*p* < 0.001): patients of African descent had the lowest TC and LDL-C levels and Indians the highest. However, there were no significant differences in high-density lipoprotein cholesterol (HDL-C) levels between ethnicities (*p* = 0.20). Adjusting for age, gender and body mass index, patients of African descent were significantly less likely to record a TC of > 4.5 mmol/l (OR 0.33, 95% CI: 0.25–0.41) compared to all ethnic groups (all *p* < 0.001).

**Conclusions:**

These data confirm important blood lipid differentials according to ethnicity in patients diagnosed with heart disease in Soweto, South Africa. Such disparities in CVD risk factors may justify the use of specialised prevention and management protocols.

## Abstract

The historical distribution of risk and communicable versus non-communicable forms of cardiovascular disease (CVD), particularly its major component heart disease, reflects the influence of cultural and ethnic factors.^1^ Historically, low levels of atherosclerotic CVD in populations of African descent were, in part, attributed to low levels of total cholesterol (TC), low-density lipoprotein cholesterol (LDL-C) and triglycerides (TGs) and high levels of high-density lipoprotein (HDL-C).^2^,^3^ Indeed, it appears a great deal of the burden of CVD in those of African descent can be attributed to hypertension, rather than dyslipidaemia.^4^ However, other ethnic groups, such as South Asians, have been shown to be more prone to the high levels of TC and TG and low HDL-C dyslipidaemia, associated with atherosclerotic forms of CVD.^5^

While a significant proportion of the excess risk of CVD in certain ethnic groups can be explained by environmental, nutritional and lifestyle factors, they do not fully account for such disparities. In order to apply appropriate CVD preventative and management strategies, it is crucial to understand the underlying processes that vary between ethnic groups, especially in settings where the burden of CVD is rapidly increasing.

In sub-Saharan African communities, such as the urban enclave of Soweto, South Africa, there is clear evidence that the historical balance between communicable and non-communicable forms of heart disease is in epidemiological transition.^6^ The Heart of Soweto study of more than 5 000 *de novo* presentations of heart disease to the Baragwanath Hospital involved patients from an eclectic mix of cultural and ethnic backgrounds and the pattern of heart disease differed accordingly.

We sought to determine if there were differences in the lipid profiles (and other major CVD risk factors) of patients with *de novo* presentations of heart disease in Soweto, South Africa according to ethnicity and whether these were independent of socio-economic profile.

## Methods

As described in detail previously,^6^,^7^ the 3 500-bed Chris Hani Baragwanath Hospital (case load of > 125 000 in-patients per annum) services the tertiary care needs of Soweto (population of 1.1 million) and surrounding communities. All cases of suspected heart disease are referred to the Hospital’s Cardiology unit for advanced diagnostic testing and gold-standard treatments. This prospective clinical registry of all *de novo* presentations (2006–2008) as part of the Heart of Soweto study^7^ represents sub-Saharan Africa’s largest and most detailed study of advanced forms of heart disease to date.^6^,^7^

The Heart of Soweto cohort of *de novo* case presentations comprised 5 328 patients. Of these, 2 185 (40%) patients simultaneously had a fasting serum TC level performed (as analysed by on-site routine chemistry laboratory). Of these, there were 1 964 cases with recorded LDL-C levels (90%), 1 996 with HDL-C levels (91%) and 1 969 with TG levels (90%). Complete lipid profiles were available for 1 945 cases (89%). None of the patients were on lipid-lowering agents at the time of presentation, as this medication would only be prescribed at the tertiary institution at the time of the study.

Overall, 518 of 5 328 *de novo* cases of heart disease were identified as HIV positive (9.7%) with 54% of these prescribed highly active anti-retroviral therapies on presentation.^8^ In this sub-study, 116 participants (5.3%) were confirmed HIV positive. Some of the patients had been placed on anti-hypertensive medication prior to their first assessment at the cardiac clinic at Baragwanath Hospital. The study was approved by the University of the Witwatersrand Ethical Committee and conforms to the principles outlined in the Declaration of Helsinki.

A complete list of study data captured by the registry, comprising basic socio-demographic (including self-reported ethnicity, years of education, and determination of birthplace as Soweto) and advanced clinical profiling has been described previously.^6^,^7^ The registry captured all advanced clinical investigative procedures (e.g. coronary angiography, which was undertaken in all people diagnosed with coronary artery disease). Echocardiography (performed on all patients) criteria used in the study have been described in detail previously.^6^,^7^

## Risk factor definition

Previous reports provide a range of thresholds for dyslipidaemia in African populations (e.g. a TC level from > 3.8 mmol/l^9^ to > 5.2 mmol/l).^10^ In this study, optimum lipid levels and treatment goals for patients with established CVD and associated conditions were defined according to European guidelines on cardiovascular disease prevention in clinical practice,^11^ as adopted by the Lipid and Atherosclerosis Society of South Africa and the South African Heart Association:^12^ high TC: > 4.5 mmol/l, high TGs: > 1.7 mmol/l and low HDL-C: < 1.0 mmol/l for males and < 1.2 mmol/l for females. Importantly, these guidelines echo the recommended LDL-C level of > 2.5 mmol/l as set by NCEP ATP III.^13^

Other risk factors for cardiovascular disease were measured, as previously described, on a clinical basis.^7^ As described in a previous report, hypertensive patients were identified on the basis of a documented blood pressure (BP) of ≥ 140/90 mmHg and/or prescribed antihypertensive treatment. In both the cardiology unit and primary care clinics, BP was assessed following five minutes’ rest; seated systolic (SBP) and diastolic (DBP) blood pressure (in mmHg) and heart rate (beats per minute) measurements were performed using an appropriately sized arm cuff via a calibrated Dynamap (Critikon, Johannesburg, South Africa) monitor. The mean of three successive readings, each separated by two minutes’ rest, was taken.

Anthropometric measurements were available for calculation of body mass index (BMI, kg/m^2^) in 1 593 (73%) cases, the low reporting rate restricted to ambulatory patients. Obesity was defined as BMI ≥ 30 kg/m^2^. Serum C-reactive protein (CRP) was measured in a sub-set of 664 patients (30% of all cases) if clinically indicated (e.g. suspected infection). Patients were stratified into risk tertiles.^14^ However, as levels of < 1.0 mg/l were not reported, we have used patients with a CRP of 1.0 mg/l (*n* = 40) as our ‘low-risk’ reference group for this parameter. This group was then compared to medium (1.1–3.0 mg/l, *n* = 50) and high (> 3.0 mg/l, *n* = 567) CRP risk categories.

Due to incomplete data to formally assess the presence of familial hypercholesterolaemia (FH) according to the Dutch Lipid Clinic Network criteria,^15^ we examined the proportion of patients with an LDL-C ≥ 4.9 mmol/l and/or TC ≥ 7.5 mmol/l to determine potential cases of FH.

## Statistical analyses

Normally distributed continuous data are presented as the mean ± standard deviation, and non-Gaussian distributed variables as the median (inter-quartile range). Categorical data are presented as percentages with 95% confidence intervals (CI) where appropriate. For group comparisons, we initially used Chi square (χ^2^) analysis with calculation of odds ratios (OR) and 95% CI (where appropriate) for discrete variables, and one-way analysis of variance (ANOVA) for normally distributed continuous variables, and Kruskal-Wallis test for non-parametric continuous variables, with Tukey’s *post hoc* tests. Multiple logistic regression analyses (entry model) were used to derive age-, gender- and BMI-adjusted ORs for the risk of presenting with dyslipidaemia.

Owing to the lower sample sizes of white Europeans, mixed-ancestry patients and Indians, these data were pooled when calculating ORs, relative to individuals of African descent. Significance was accepted at the two-sided level of *p* < 0.05 and *p* < 0.01 (Bonferroni correction) for ANOVA and Kruskal-Wallis analyses.

## Results

Table 1 shows the socio-demographic and clinical profiles of this cohort according to ethnicity. While there were no differences in mean age across ethnicities, there were significant differences in the proportion of females; ranging from 61% in those of African descent to 36% in white Europeans. Overall, compared to all other ethnicities, African patients were more likely to be obese (OR 1.56, 95% CI: 1.15–2.13, *p* < 0.01 in age- and gender-adjusted model) and have fewer than six years’ education (OR 1.72, 95% CI: 1.28–2.31, *p* < 0.0001).

**Table 1.** Clinical And Demographic Profile According To Ethnicity

	*African descent (n = 1823)*	*White European (n = 142)*	*Mixed ancestry (n = 87)*	*Indian (n = 133)*	p*-value*
Demographic profile					
Mean age (years)	56.2 ± 15.8	57.3 ± 12.7	55.7 ± 12.1	58.6 ± 11.9	0.29
Female	1104 (61%)	51 (36%)	35 (40%)	68 (51%)	< 0.001
< 6 years’ formal education	856 (47%)	33 (23%)	31 (36%)	71 (53%)	< 0.001
Soweto origin	856 (47%)	2 (1%)	2 (2%)	0 (0%)	< 0.001
Clinical presentation					
Total cholesterol (mmol/l)	4.1 ± 1.3	4.6 ± 1.3***	4.8 ± 1.3***	5.0 ± 1.1***^	< 0.001
Low-density lipoprotein cholesterol (mmol/l)	2.4 ± 1.0	2.7 ± 1.0*	2.8 ± 1.1*	3.0 ± 1.0***	< 0.001
High-density lipoprotein cholesterol (mmol/l)	1.2 ± 0.5	1.2 ± 0.5	1.2 ± 0.5	1.2 ± 0.4	0.24
Median triglycerides (mmol/l)	1.1 (0.8–1.6)	1.4 (1.0–2.1) ***	1.4 (0.9–1.9)**	1.8 (1.2–2.7) ***^^#^	< 0.001
Median serum CRP (*n* = 664, mg/l)	16.6 (6.2–83.9)	27.0 (8.0–98.0)	25.0 (6.3–50.2)	7.9 (2.7–27.0)	0.03
Systolic blood pressure (mmHg)	136.6 ± 28.2	130.1 ± 27.1*	133.7 ± 25.9	133.8 ± 24.0	0.05
Diastolic blood pressure (mmHg)	77.8 ± 15.4	72.8 ± 12.8 *	75.8 ± 15.0	73.7 ± 12.2*	< 0.001
Body mass index (*n* = 1593, kg/m^2^)	29.7 ± 7.5	29.7 ± 9.8	25.6 ± 7.1*^^	28.2 ± 5.7	< 0.001
Prevalence of dyslipidaemia					
High total cholesterol (mmol/l)	715 (39%)	76 (54%)	52 (60%)	93 (70%)	< 0.001
High low-density lipoprotein cholesterol (mmol/l)	718 (44%)	68 (55%)	47 (60%)	85 (72%)	< 0.001
High triglycerides (mmol/l)	386 (23%)	44 (36%)	33 (41%)	66 (56%)	< 0.001
Low high-density lipoprotein cholesterol (mmol/l)	1044 (63%)	86 (66%)	47 (57%)	74 (62%)	0.58
Prevalence of other risk factors					
Obese (> 30 kg/m^2^)	579 (43%)	31 (37%)	12 (19%)	29 (30%)	< 0.001
Type 2 diabetes	164 (9%)	9 (6%)	6 (7%)	18 (14%)	0.34
Current smoker	833 (46%)	104 (73%)	63 (72%)	57 (43%)	< 0.001
Family history of CVD	765 (42%)	71 (50%)	34 (39%)	88 (66%)	< 0.001
Primary diagnosis					
Hypertension	380 (21%)	15 (11%)	23 (26%)	33 (25%)	0.42
Hypertensive heart failure	525 (29%)	14 (10%)	14 (16%)	22 (17%)	< 0.001
Coronary artery disease	179 (10%)	74 (52%)	23 (26%)	61 (46%)	< 0.001

CRP = C-reactive protein; CVD = cardiovascular disease; median (interquartile range); Kruskal-Wallis test performed. Tukey’s *post-hoc* tests: *p < 0.05; **p < 0.01; ***p < 0.001 compared to African descent. ^*p* < 0.05; ^^*p* < 0.01 compared to white Europeans; ^##^*p* < 0.01 compared to mixed ancestry.

White European patients recorded the lowest prevalence of poor education (23%) but higher rates of smoking; when compared to patients of African descent, white Europeans were three-fold more likely to be current smokers (OR 3.4, 95% CI: 1.86–5.64, *p* < 0.001). Patients of mixed ancestry were twice as likely to be current smokers than patients of African descent (OR 1.91, 95% CI: 1.08–3.36, *p* = 0.03).

Indian patients were most likely to report a family history of heart disease (66% of patients, OR 3.96, 95% CI: 2.46–6.37, *p* < 0.001 compared to African descent). There were no significant differences between ethnicities in diabetes co-morbidity, despite a relatively high prevalence in Indian patients (14% compared to 9% in all other ethnic groups; *p* = 0.08).

African patients were significantly more likely to be diagnosed with hypertensive heart failure (HT-HF) (OR 2.14, 95% CI: 1.48–3.11) and least likely to be diagnosed with coronary artery disease (CAD) (OR 0.16, 95% CI: 0.12–0.22, *p* < 0.001 for both). Despite representing only 12% of patients in this sub-study, white Europeans and Indian patients accounted for 40% of all primary diagnoses of CAD.

## Lipid profiles

**Fig. 1.**
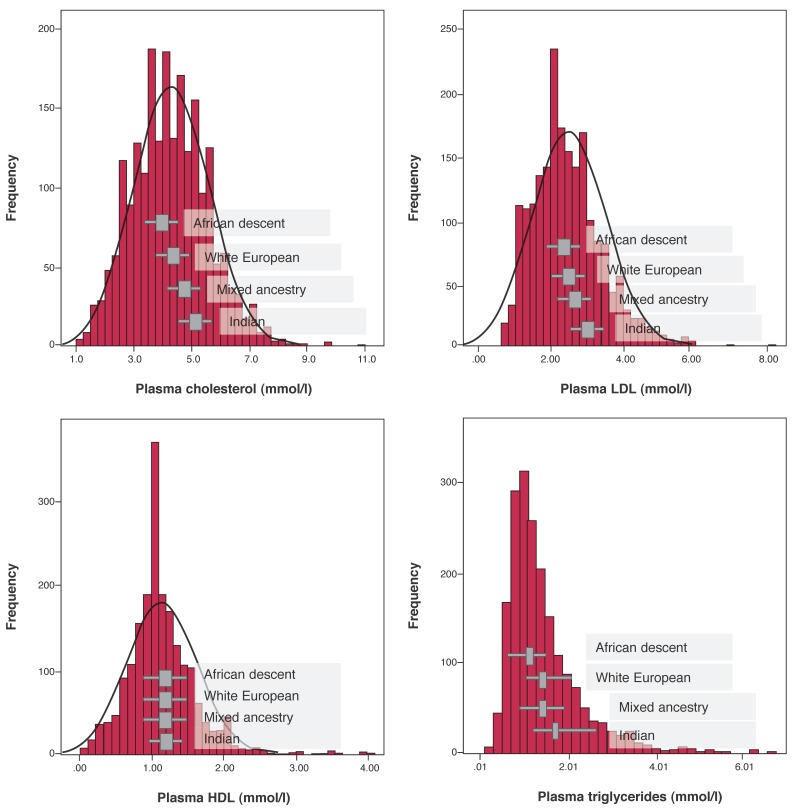
Frequency distribution of lipid levels according to ethnicity. Lipid levels (mmol/l): mean (± standard deviation) in A, B and C. Median (inter-quartile range) for triglycerides (D). TC = total cholesterol; LDL = low-density lipoprotein cholesterol; HDL = high-density lipoprotein cholesterol.

There were significant (unadjusted) differences in lipid profiles across ethnicities with increasing gradients in TC, LDL-C and TG levels (all *p* < 0.001), however there were no differences observed between ethnicities in HDL-C values (*p* = 0.24) (Fig. 1). Overall, females had higher HDL-C values than males: 1.21 ± 0.50 vs 1.09 ± 0.50 mmol/l (*p* < 0.0001). While females had higher HDL-C levels than males in the African (*p* < 0.001), white European (*p* = 0.04) and Indian (*p* = 0.01) groups, there was no difference in HDL-C levels in those of mixed ancestry: 1.33 ± 0.42 mmol/l in females vs 1.19 ± 0.60 mmol/l in males (*p* = 0.23).

When compared to other ethnicities (pooled analyses), patients of African descent had significantly lower TC, LDL-C and TG levels (*p* < 0.01 for all comparisons). African patients were significantly less likely to have high TC (OR 0.33, 95% CI: 0.25–0.44), high LDL-C (OR 0.34; 95% CI: 0.25–0.47) and high TG levels (OR 0.37, 95% CI: 0.27–0.50) compared to other ethnicities (all *p* < 0.001).

In gender- and age-adjusted linear regression models, BMI was positively but modestly associated with TC, LDL-C and TG levels (all *p* < 0.01). The estimated mean increase in TC level was 0.09 mmol/l (95% CI: 0.01–0.85; *p* < 0.0001) for a 5-kg/m^2^ increase in BMI, while LDL-C increased 0.05 mmol/l for the same change in BMI (95% CI: 0.03–0.1, *p* = 0.002). There was also a positive association between TG values and BMI; a 5-kg/m^2^ increase in BMI was associated with a mean 0.07-mmol/l increase in TG level (95% CI: 0.04–0.1, *p* < 0.001). There was no association between BMI and HDL-C level (*p* = 0.8).

Table 2 shows the independent predictors of dyslipidaemia in this cohort. Female gender was a positive predictor for high TC levels and females were less likely to have a low HDL-C value. Patients with poor education (< six years of formal education) were more likely to have lower lipid levels. The effect of urban upbringing versus migration (typically rural to urban) on dyslipidaemia in this cohort was not apparent; Soweto origin was not related to high TC and LDL-C or low HDL-C levels.

**Table 2.** Independent Correlates Of High Serum Total Cholesterol, High Low-Density Lipoprotein And Low High-Density Lipoprotein Cholesterol

	*High TC*		*High LDL-C*		*Low HDL-C*	
	*Odds Ratio*	*95% CI*	*Odds Ratio*	*95% CI*	*Odds Ratio*	*95% CI*
Socio-demographic profile						
Female gender	1.39	1.12–1.72*	1.26	0.98–1.62	0.59	0.46–0.74**
Age	1.00	1.00–1.01	1.00	1.00–1.01	0.99	0.98–0.99**
< 6 years’ formal education	0.68	0.56–0.84**	0.74	0.59–0.92*	1.56	1.24–1.96**
Soweto origin	0.83	0.67–1.02	0.86	0.69–1.07	0.93	0.74–1.15
Diabetes comorbidity	1.0	0.72–1.38	0.99	0.70–1.40	1.23	0.86–1.77
Current smoker	0.72	0.58–0.89**	0.68	0.54–0.85**	1.47	1.17–1.85**
Ethnicity						
African ancestry (reference)	1.0		1.0		1.0	
White European	2.17	1.36–3.46*	2.10	1.27–3.47*	1.01	0.61–1.68
Mixed ancestry	3.04	1.79–5.15**	2.44	1.41–4.22*	0.71	0.42–1.20
Indian	4.15	2.61–6.60**	4.66	2.72–7.97**	1.00	0.63–1.59

TC = total cholesterol; LDL-C = low-density lipoprotein cholesterol; HDL-C = high-density lipoprotein cholesterol; CI = confidence intervals. Age-, gender- and body mass index-adjusted analysis: *p < 0.01; **p < 0.001.

However the effect of ethnicity on dyslipidaemia was apparent: compared to patients of African descent, white European, mixed-ancestry and Indian patients were two-, three- and four-fold more likely to have high TC levels, respectively. These lipid gradients, ranked across ethnicities, persisted for high LDL-C and high TG levels in age-, gender- and BMI-adjusted models; white European (OR 1.58, 95% CI: 0.92–2.69; *p* = 0.10), mixed-ancestry (OR 2.57, 95% CI: 1.50–4.39; *p* = 0.001) and Indian (OR 4.33 95% CI: 2.75–6.83; *p* < 0.0001) patients were all more likely to have high TG levels compared to patients of African descent. Ethnicity was not associated with low HDL-C levels (Table 2). While current smokers were 1.5-fold more likely to have low HDL-C levels (OR 1.47, 95% CI: 1.17–1.85, *p* = 0.001), diabetes co-morbidity was not associated with low HDL-C (*p* = 0.3).

CRP was only measured in a limited number of patients. However, subgroup analysis showed that serum CRP was strongly associated with low HDL-C: in age- and genderadjusted models, patients in the medium- (OR 3.17, 95% CI: 1.10–9.12; *p* = 0.03) and high-risk CRP groups (OR 5.58, 95% CI: 2.34–13.31; *p* < 0.001) were more likely to have low HDL-C levels compared to the low-risk reference group (i.e. those with a CRP of 1.0 mg/l). There was no association between CRP and high TC, LDL-C or TG levels.

Overall, 43 patients (2.2% of cohort with LDL-C levels measured) recorded an LDL-C ≥ 4.9 mmol/l, and 32 patients (1.8% with TC levels measured) had TC ≥ 7.5 mmol/l. A total of 56 patients (2.6%), comprising 43 of African descent (2.4% of ethnic group), six white Europeans (4.2%), three of mixed ancestry (3.4%) and four Indians (3.0%) had either elevated LDL-C or TC levels, suggestive of FH.

## Discussion

This study provides important insights into the lipid profiles according to ethnicity of more than 2 000 *de novo* cases of heart disease in Soweto, South Africa. We found significant gradients in lipid levels on this basis. Patients of African descent had the lowest, and Indians the highest TC, LDL-C and TG levels. These gradients in TC and LDL-C levels were particularly distinct in their magnitude and may represent clinically important differences for the development of atherosclerosis (see below). However, HDL-C levels did not differ across ethnic groups. Therefore the assumed protective role of HDL-C in the African population was not evident beyond cases of CAD (where African patients were under-represented).

Overall, there were important differences in the risk factor and clinical profile of cases according to ethnicity. Irrespective of the relatively low levels of TC, low HDL-C was widespread, affecting nearly two-thirds (63%) of this cohort. This was surprising when considering the proportion affected with high TC (43%), LDL-C (47%) and TG (27%) levels. As a strong, independent predictor of CVD,^16^ this phenomenon of low HDL-C levels represents a potentially important therapeutic target for truncating an increasing burden of atherosclerotic disease in urban, sub-Saharan African communities. It is possible that an acute-phase response could lower HDL-C levels in most instances and may account for the reciprocal relationship found between CRP and HDL-C.

Our observations in respect of ethnic differentials in lipid profiles are broadly consistent with prior studies in the region.^9^,^17^-^20^ Previous research has shown that those of African descent display an athero-protective lipid profile compared to white Europeans and Indians.^5^ Similarly, the INTERHEART Africa case–control study (which investigated risk factors associated with first-time myocardial infarction) showed no difference in HDL-C levels between those of African descent, mixed-ancestry and European/other African groups.^18^ However, fewer than 200 cases were of African descent.

Norman and colleagues reported in 2000 that cholesterol-attributable mortality rates were highest in the Indian population of South Africa (at 22.2%), followed by white European (20.5%) and mixed-ancestry (8.8%) populations.^9^ By comparison, mortality attributable to ‘sub-optimal’ lipid levels (defined as TC ≥ 3.8 mmol/l) in those of African descent was only 1.8%, which reflected low TC levels compared to other ethnicities.^9^ Nonetheless, varied reports show that dramatic increases in mean TC levels have occurred in the past four decades,^19^,^20^ while HDL-C levels have decreased,^21^,^22^ both of which may be contributing to the increase in CAD prevalence over a similar time period.^2^ It is likely that the continued influences of epidemiological transition (giving rise to extensive urbanisation, Western diet and sedentary lifestyle) will continue to fuel these patterns.^23^

The prevalence of FH in African populations is unknown but is probably about 1:500, as has been reported in most other populations studied worldwide. South Africa has several communities in which there are founder effects that increase the prevalence of FH, including the Afrikaner, Jewish and Indian communities. Several LDL-C receptor errors have been identified in patients of mixed ancestry.^24^ In this cohort, genetic disorders such as FH were unlikely to be a major contributor to *de novo* presentations of heart disease, given the small number of probable/possible cases of the same (2.3%). Mixed hyperlipidaemia that could relate to an uncommon mutation in apoE in those of African descent was also not suspected.

Examining the potential impact of migration on CVD and its risk factors may be helpful in understanding ethnic differences in the relative incidence of CVD in different African populations.^25^ In our multivariate analyses, urban (Soweto) origin was not associated with high TC or LDL-C, or low HDL-C levels. This was similar to a study on adults of the Cape Peninsula,^26^ in which measures of urbanisation had no effect on TC levels *per se*; rather, it was clusters of risk factors that corresponded with increased time residing in an urban setting.

We identified more than six years of formal education as being independently predictive of high TC and LDL-C, and low HDL-C levels, which corresponds to other findings from the Heart of Soweto study.^6^ These results may indicate a progression in income/socio-economic status, enabling greater access to a Western diet and increased tendency for sedentary behaviour,^27^ especially in women.^23^ However, we have shown that the ethnic-based patterns of dyslipidaemia presented in this report persisted even after controlling for BMI. This important finding alludes to the influence of other factors that contribute to the presentation of heart disease and its risk factors.

Other factors, such as very low TG levels,^3^ and novel factors unrelated to dyslipidaemia (such as more effective homocysteine metabolism),^28^ may confer relative protection against atherosclerotic heart disease in African populations. Patterns of hypertension and obesity, which persist even in migrated African populations,^3^ may suggest predisposing factors. However, the case for attributing genetic susceptibility to heart disease solely on the basis of ‘race/ethnicity’ is proving increasingly futile.^29^

In this study, we could clearly discount monogenic disorders of lipoproteins as the cause of lipid differentials across ethnicities, as they were uncommon. We have seen features of dyslipidaemia change in these populations over time,^20^-^22^ especially with regard to HDL-C levels. It is more likely that risk factors that can influence lipids, particularly obesity and its antecedents (poor diet and lack of physical activity),^23^ are driving the patterns of dyslipidaemia reported here. Relying on genetic susceptibility to explain ethnic differences in lipids, without recognising the role of gene–environment interaction in CVD risk will only prove counterproductive in addressing current prevention and management of CVD.

Given that dyslipidaemia does not occur in isolation, future work should focus on likely contributors to these trends, irrespective of ethnicity. Insulin resistance or the metabolic syndrome dyslipidaemia is strongly associated with atherosclerotic CVD and may play a role in the consistently low HDL-C levels across ethnic groups, as reported here. In Indian patients, the prevalence of diabetes was 18%, which may contribute to the high rates of CAD in this group (half of primary diagnoses in those of Indian ethnicity), an association that has been shown previously.^5^ Despite a lower prevalence of diabetes in other ethnicities, overall high obesity rates in this cohort will likely contribute to an increased prevalence of both insulin resistance and diabetes in coming generations.^23^,^30^

Additionally, we found an inverse association between the inflammatory marker CRP and HDL-C levels in those whom the inflammatory marker was measured. Despite the subset representing only 30% of those with a reported lipid profile, the negative association was striking and may indicate enhanced CVD risk beyond other clinical risk factors.^14^ Therefore, the possible contribution of chronic, low-grade inflammation to dyslipidaemia warrants additional investigation, as it is associated with both chronic infection and atherosclerotic CVD.^14^

There are a number of limitations that require consideration. Clinical data (other than routine echocardiography and 12-lead ECG) were obtained according to clinical presentation and only those with suspected atherosclerotic disease had lipid levels measured. Systematic bias, therefore, needs to be carefully considered before attributing broad patterns in lipid profiles according to ethnicity and may overestimate the utility of measuring/treating dyslipidaemia within the wider community.

Adiposity (BMI), a major confounder of both dyslipidaemia and heart disease, was recorded in 73% of patients in this cohort. However, its inclusion in the regression analyses did not alter the significant findings. Central obesity measurements (e.g. waist-to-hip ratio) may have offered greater delineation of CVD risk, given its strong association with low HDL-C levels, particularly in women of African descent,^10^ however this was an impractical variable to obtain consistently, given the nature of the study setting. CRP was only measured in around one-third of selected cases and related data require careful interpretation.

## Conclusion

Differences in CVD risk factors, especially lipid profiles, were apparent in the ethnically diverse enclave of Soweto in South Africa. We have shown that while dyslipidaemia is an important risk factor in heart disease presentation, overall, disparities exist in the extent of lipid abnormalities. Significantly, low HDL-C levels persisted in all ethnicities. These trends have important public health and clinical implications that require further consideration. While there are inherent limitations in the interpretation of racial and ethnic comparisons, irrespective of the healthcare setting, rational approaches to secondary prevention of heart disease may require a diversity of strategies because of these ethnic differences.

## References

[1] Yusuf S, Reddy S, Ounpuu S, Anand S (2001). Global burden of cardiovascular diseases: Part II: variations in cardiovascular disease by specific ethnic groups and geographic regions and prevention strategies.. Circulation.

[2] Akinboboye O, Idris O, Akinkugbe O (2003). Trends in coronary artery disease and associated risk factors in sub-Saharan Africans.. J Hum Hypertens.

[3] Zoratti R (1998). A review on ethnic differences in plasma triglycerides and high-density-lipoprotein cholesterol: is the lipid pattern the key factor for the low coronary heart disease rate in people of African origin?. Eur J Epidemiol.

[4] Addo J, Smeeth L, Leon DA (2007). Hypertension in sub-saharan Africa: a systematic review.. Hypertension.

[5] Cappuccio FP (1997). Ethnicity and cardiovascular risk: variations in people of African ancestry and South Asian origin.. J Hum Hypertens.

[6] Stewart S, Carrington M, Pretorius S, Methusi P, Sliwa K (2011). Standing at the crossroads between new and historically prevalent heart disease: effects of migration and socio-economic factors in the Heart of Soweto cohort study.. Eur Heart J.

[7] Sliwa K, Wilkinson D, Hansen C, Ntyintyane L, Tibazarwa K, Becker A (2008). Spectrum of heart disease and risk factors in a black urban population in South Africa (the Heart of Soweto Study): a cohort study.. Lancet.

[8] Sliwa K, Carrington MJ, Becker A, Thienemann F, Ntsekhe M, Stewart S (2011). Contribution of the human immunodeficiency virus/acquired immunodeficiency syndrome epidemic to de novo presentations of heart disease in the Heart of Soweto Study cohort.. Eur Heart J.

[9] Norman R, Bradshaw D, Steyn K, Gaziano T (2007). Estimating the burden of disease attributable to high cholesterol in South Africa in 2000.. S Afr Med J.

[10] Njelekela MA, Mpembeni R, Muhihi A, Mligiliche NL, Spiegelman D, Hertzmark E (2009). Gender-related differences in the prevalence of cardiovascular disease risk factors and their correlates in urban Tanzania.. BMC Cardiovasc Disord.

[11] Graham I, Atar D, Borch-Johnsen K, Boysen G, Burell G, Cifkova R (2007). European guidelines on cardiovascular disease prevention in clinical practice: executive summary: Fourth Joint Task Force of the European Society of Cardiology and Other Societies on Cardiovascular Disease Prevention in Clinical Practice (Constituted by representatives of nine societies and by invited experts).. Eur Heart J.

[12] Butler N (2009). National Guidelines at a glance: Hypercholesterolaemia.. SA Pharmaceut J.

[13] (2002). Third Report of the National Cholesterol Education Program (NCEP) Expert Panel on Detection, Evaluation, and Treatment of High Blood Cholesterol in Adults (Adult Treatment Panel III) final report.. Circulation.

[14] Pearson TA, Mensah GA, Alexander RW, Anderson JL, Cannon RO, Criqui M (2003). Markers of inflammation and cardiovascular disease: application to clinical and public health practice: A statement for healthcare professionals from the Centers for Disease Control and Prevention and the American Heart Association.. Circulation.

[15] Civeira F (2004). Guidelines for the diagnosis and management of heterozygous familial hypercholesterolemia.. Atherosclerosis.

[17] Chapman MJ, Ginsberg HN, Amarenco P, Andreotti F, Boren J, Catapano AL (2011). Triglyceride-rich lipoproteins and high-density lipoprotein cholesterol in patients at high risk of cardiovascular disease: evidence and guidance for management.. Eur Heart J.

[17] Maritz FJ (2006). Dyslipidaemia in South Africa..

[18] Steyn K, Sliwa K, Hawken S, Commerford P, Onen C, Damasceno A (2005). Risk factors associated with myocardial infarction in Africa: the INTERHEART Africa study.. Circulation.

[19] Seftel HC, Asvat MS, Joffe BI, Raal FJ, Panz VR, Vermaak WJ (1993). Selected risk factors for coronary heart disease in male scholars from the major South African population groups.. S Afr Med J.

[20] Truswell AS, Mann JI (1972). Epidemiology of serum lipids in Southern Africa.. Atherosclerosis.

[21] Oelofse A, Jooste PL, Steyn K, Badenhorst CJ, Lombard C, Bourne L (1996). The lipid and lipoprotein profile of the urban black South Africa population of the Cape Peninsula – the BRISK study.. S Afr Med J.

[22] Walker AR, Walker BF (1978). High high-density-lipoprotein cholesterol in African children and adults in a population free of coronary heart diseae.. Br Med J.

[23] Kruger HS, Puoane T, Senekal M, van der Merwe MT (2005). Obesity in South Africa: challenges for government and health professionals.. Public Health Nutr.

[24] De Villiers WJ, van der Westhuyzen DR, Coetzee GA, Henderson HE, Marais AD (1997). The apolipoprotein E2 (Arg145Cys) mutation causes autosomal dominant type III hyperlipoproteinemia with incomplete penetrance.. Arterioscler Thromb Vasc Biol.

[25] Vorster HH (2002). The emergence of cardiovascular disease during urbanisation of Africans.. Public Health Nutr.

[26] Steyn K, Kazenellenbogen JM, Lombard CJ, Bourne LT (1997). Urbanization and the risk for chronic diseases of lifestyle in the black population of the Cape Peninsula, South Africa.. J Cardiovasc Risk.

[27] Pearson TA (2003). Education and income: double-edged swords in the epidemiologic transition of cardiovascular disease.. Ethn Dis.

[28] Ubbink JB, Vermaak WJ, Delport R, van der Merwe A, Becker PJ, Potgieter H (1995). Effective homocysteine metabolism may protect South African blacks against coronary heart disease.. Am J Clin Nutr.

[29] Goodman AH (2000). Why genes don’t count (for racial differences in health).. Am J Public Health.

[30] Shaw JE, Sicree RA, Zimmet PZ (2010). Global estimates of the prevalence of diabetes for 2010 and 2030.. Diabetes Res Clin Pract.

